# Chicago Medical Society

**Published:** 1882-02

**Authors:** 


### ARTICLE IX.

CHICAGO MEDICAL SOCIETY. Stated meeting, Jan. 16, 1882.
Dr. E. Ingals, President, in the Chair.

Dr. L. H. Montgomery read a lengthy report of the preceding meeting, which was accepted after a few corrections. These reports of the Secretary are intended to give a *résumé* of all papers which have come before the society, and are very valuable.

Mr. Stanton, of the *Chicago Medical Review*, read the paper announced for that evening, on

### OPENING AND DRAINAGE OF JOINTS, by Drs. Chr. Fenger and E. W. Lee.

From the earliest times, suppurative arthritis had been looked upon as a very serious disease, which sometimes caused the death of the patient, and generally left permanent injury. There were cases on record in which a spontaneous opening of the cavity of a joint, with escape of the pus, had been followed by recovery. However, although thus indicated, the means at hand did not legitimate the drainage of such suppurating cavities before the introduction of Dieulafoy's aspirator.

It was found, however, that aspiration, beneficial in a few cases, was useless in a majority of them, and the antiseptic method of treating abscess promised far better results. In reporting the following cases, the authors of the paper did not believe that they could give anything new to the profession, since similar cases had already been reported; but they believed that a better acquaintance with their results would induce other surgeons to follow it, and, in future, we could entirely dispense with amputation in such cases, and, if opened early, the chances were that a certain amount of motion at least would be insured; but they considered the use of the spray, and other particulars of the method taught by Lister, a *sine quâ non* for a successful operation.

More than ten years ago, five cases of drainage of the joints had been reported, and since that, a few sporadic cases had appeared in medical papers at various times. The most superficial joints, that is, those that were of easiest access, naturally suggested the first trials; as the elbow, the knee and the ankle. Only a few of the other joints had ever been opened. In the knee, a diagnosis was easy, and a slight amount of fluctuation could be detected. Two of their cases were of that joint.

*Case* I.—A. S., female; admitted to the County Hospital May 30, 1879. Two weeks before admission, inflammation, with swelling of the knee, had manifested themselves, with an intense pain, which went on increasing, and fever set in. June 5, her temperature was 103°. Some pus had been aspired from the knee, and June 16 an elastic bandage was applied. June 29, joint was aspired once more, and July 1 the temperature was 105.2, and vomiting occurred.

The patient having been anæsthetized, Dr. Fenger made an incision in either side of the patella, passed in a fenestrated rubber drainage tube, which was retained in position by sutures, and washed the cavity of the knee with a solution of 2.5 per cent. of carbolic acid. The temperature fell, the pain was relieved, and re-dressing was done July 4; all in accordance with the antiseptic method. Patient improved from the day of the operation. July 21, the drainage tube was removed, and the wound allowed to heal. October 3, the plaster cast was applied to immobilize the limb, and the patient obtained about as good a leg as the sound one, with a fair amount of motion. The patient, two weeks after the operation, had thronbosis of femoral vein, which gave rise to a high fever; and later she had a diarrhœa which retarded her final recovery.

June 5, 1880, she was quite well.

*Case* II.—E. M., female; had acute rheumatism, followed with a suppurative inflammation of the knee-joint. Aspiration was resorted to twice without marked results, and it was decided to operate.

July 14, the patient having taken ether, the knee was opened on either side of the patella under the spray, and irrigation was made through a drainage tube, in the same manner as in the first case. July 16, it was re-dressed, and the outer half of the drainage tube was removed, and the rest of it was taken away July 19. August 7, the limb could be bent at right angle; November 22, this leg was almost as strong as the other one. In this case there had been hardly any fever during the treatment.

The operation in such case should be made in the following manner:

Make a short incision half an inch from the border of the patella, and pass a probe through the incision and through the joint to the other side, about half an inch from the border of the patella on that side; then make another incision, cutting on the point of the probe. Pass the drainage tube from one incision to the other; wash the cavity with a solution of carbolic acid, 2.5 per cent., and attach safety pins to the ends of the drainage tube, after soaking in a solution of carbolic acid. Then, after dressing the knee antiseptically, suspend it in a fenestrated splint. Re-dress as often as necessary, under the spray, and remove the drainage tube after no new pus is formed. Then put the limb in a plaster cast, let the patient get a high soled shoe for the healthy limb, and let him go about on crutches. After five weeks, remove the plaster cast.

Operations on the ankle joint are not so favorable, partly because the capsule of the joint forms two pouches, one anteriorly and the other posteriorly.

*Case* I.—N. B., æt. thirty-seven, came under treatment July 20, 1880. Had suffered from poli-articular rheumatism, and had suppurative inflammation of the left ankle joint.

July 25, the pain was intense, and other symptoms rendered the operation imperative. After administering ether, two incisions were made on either side of the joint, anteriorly to the malleoli; the cavity was washed, the drainage tube and dressing applied. The drainage tube was removed after six weeks. September 15 the opening had healed. There was some stiffness left.

*Case* II.—P. G. B., æt. twenty-two; male. In April, 1879, a plank struck his left ankle. The injury passed away; but three months later, pain, swelling, and inflammation set in. He was admitted to hospital in September.

November 15, the joint was opened in the manner above described, and the drainage of the cavity insured. There was no denuded bone found. The drainage tube was removed in a week, but had to be re-inserted December 19 for two weeks. Six months later there was limited flexion, but the limb could not be used. Six months after this, the patient was yet unable to walk without crutches, and there was necessity for another operation, but he would not submit to it.

*Case* III.—A. S. had rheumatic inflammation of ankle joint, with suppuration, and was operated on September 25, with all the precautions described in regard to the other cases. October 8 the tube was removed in part, and the rest of it taken away October 15. October 24, another incision was necessary, and the drainage tube, then re-inserted, was afterward removed November 24. It had to be re-inserted in January. Further particulars of the case will be reported later.

Notice that in such cases, the life of the patient is sometimes saved by the operation. The fever and the pain immediately abate, and improvement follows. In operating on the ankle, the doctor believed that two drainage tubes and four openings, two in front and two behind the malleoli, would answer better than the method here followed. It would involve no additional danger, if made under the spray.

An early operation saved some mobility, and prevented a disintegration of the articular extremities, which, after it had taken place, would be very likely to cause some anchylosis.

DISCUSSION.

Dr. Roswell Park asked whether, in cases of chronic synovitis, with sero-fibrinous exudation, without pus, and in which repeated injections of 20 mm. of a 2.5 per cent. carbolic acid solution had been recommended—whether in such cases drainage of the joints was ever indicated?

Dr. Fenger said that in such cases the operation was not so imperative; yet he would not hesitate to open into the joint if repeated aspirations failed of curing the arthritis. However, it was mainly in cases where the cavity contained pus that drainage became necessary. This, made with antiseptic precautions, he considered devoid of danger. After opening into a joint, the doctor said it was always important to ascertain that no communication existed between it and some other cavities, and it was always better to make a counter-opening.

Dr. R. Tilley mentioned the fact that some of the most successful surgeons in Europe had discarded the use of the spray, and even that of carbolic acid, and had obtained as good results without it. He wished to have the opinion of some of the members present.

Dr. Fenger would not abandon the use of carbolic acid, because he had been so satisfied of the results obtained with it; no substitute could take its place, and the danger of pyæmia, always present in hospitals, could be avoided by its use. It was a fact that the best surgeons used it, and that the best results claimed for it were in connection with the filthiest hospitals. Advices to the contrary he considered as unreliable. It was true that Lawson Tait had obtained good results in ovariotomy without the use of the antiseptic method; but he did not consider that in such cases there was as much tendency to suppuration as in almost any other class of operations.

Dr. E. W. Lee, one of the authors of the paper, said that, although an admirer of Lister's method, he was obliged to abandon the spray in self-defense five months ago. The results obtained with it had always been satisfactory, but it affected his health, and every operation prostrated him. The spray sometimes gave away, or some other particular of the method was neglected, and this accounted for failure in the results, which otherwise were very satisfactory. He did not believe that any other method could be substituted for it, and that where free incisions were made without antiseptic precautions, there was always more suppuration, and a necessity for more frequent dressings. The doctor alluded to the packing of suppurative cavities with iodoform, but he believed some degree of poisoning had followed its use in many cases.

